# An investigation of maternal anaemia among HIV infected pregnant women on antiretroviral treatment in Johannesburg, South Africa

**DOI:** 10.11604/pamj.2020.37.93.22244

**Published:** 2020-09-25

**Authors:** Jewelle Methazia, Emery Ladi Ngamasana, Wells Utembe, Modupe Ogunrombi, Peter Nyasulu

**Affiliations:** 1 Ibis Reproductive Health, Parktown, Johannesburg, South Africa,; 2 Division of Epidemiology and Biostatistics, Department of Global Health, Faculty of Medicine and Health Sciences, Stellenbosch University, Cape Town, South Africa,; 3 National Institute for Occupational Health, National Health Laboratory Services, Johannesburg, South Africa,; 4 Sefako Makgatho Health Sciences University, Pretoria, South Africa,; 5 Division of Epidemiology and Biostatistics, School of Public Health, Faculty of Health Sciences, University of the Witwatersrand, Johannesburg, South Africa

**Keywords:** Anaemia, HIV, pregnancy, ART, Themba Lethu Clinic, Johannesburg

## Abstract

**Introduction:**

maternal anaemia is a major public health problem in developing countries. Data suggests that anaemia contributes to the progression of Human Immunodeficiency Virus (HIV)-infection. The aim of this study was to investigate if pregnancy was an aggravating factor for anaemia among HIV-positive women on anti-retroviral treatment (ART).

**Methods:**

we analyzed data of all HIV-positive women aged 18-49 years receiving ART at Themba Lethu Clinic, Helen Joseph Hospital, Johannesburg, South Africa, from 1^st^ April 2004- 30^t^ ^h^April 2011. HIV-positive pregnant women were matched with non-pregnant women using the year of initiation of treatment. The outcome of interest ´anaemia´ was defined as “no anaemia”, “anaemia” and “moderate/severe anaemia”. We fitted an ordered logistic regression model to predict the likelihood of having severe/moderate anaemia versus no anaemia. We included pregnancy status as a predictor of the outcome and controlled the effect of other covariates in the analysis.

**Results:**

the study included 236 HIV positive patients, of which half (n=118, 50%) were pregnant. At baseline, about (n=143, 60%) of patients were anaemic. The proportion of pregnant women classified as anaemic (anaemia, moderate/severe) differed significantly (p=0.02) from that of non-pregnant women. The following characteristics were significantly associated with anaemia at baseline: Body mass index (BMI) category (p=0.01); World Health Organization (WHO) stage (p=0.001) and CD4 count (p=0.001). Seven months after initiation of treatment, the proportion of HIV positive women with anaemia decreased significantly.

**Conclusion:**

anaemia is a significant risk factor for untoward health outcomes, especially among HIV-positive pregnant women. Early ART access might result in a significant decrease in anaemia in pregnancy.

## Introduction

Anaemia occurs when the oxygen-carrying capacity of red blood cells is insufficient and fails to satisfy the physiologic demands of the body [1]. Most disability-adjusted life years (DALYs) caused by anaemia occur in low-income countries, representing approximately 3.9% of DALYS in women in sub-Saharan Africa (SSA) [2]. Anaemia has become a common clinical manifestation in Human Immunodeficiency Virus (HIV) infection and is found in an estimated 30% of patients with HIV and as much as 75-80% of those diagnosed with Acquired Immunodeficiency Syndrome (AIDS) [3]. In 2017, the HIV prevalence in South Africa (SA) was 13.1% translating to approximately 7.9 million people [4].

The causes of anaemia in HIV infected patients are multifactorial and include the direct effects of the infection and its viral proteins, coupled with immune dysregulations in HIV infection, which can be responsible for bone marrow suppression, as well as the presence of pathogenic opportunistic infections that causes abnormalities in blood cell counts [5]. The potential causes of anaemia within the context of HIV include gastrointestinal blood loss, malignancies, bone marrow infections, deficiency of erythropoietin, immune system-mediated haemolysis and the HIV infection of the haematopoietic stem cell [6]. Independent of viral load and CD4 counts, the incidence of anaemia can influence the progression of HIV to AIDS whilst the recovery from anaemia significantly reduces the risk of death and improves the survival of those infected with HIV [6,7].

Anaemia is also common among pregnant women and studies in SA have reported an extremely high prevalence in HIV infected pregnant women with high rates of perinatal and maternal morbidity [2,7-9]. Existing evidence shows the significant difference in the incidence of anaemia among HIV infected pregnant women at booking compared to those who have already been on anti-retroviral treatment (ART) [8]. In SA, this is important since 30% of pregnant women attending public health care facilities were reported to be HIV positive [10]. Anaemia during pregnancy is defined as a haemoglobin concentration of less than 11.0g/dl, while moderate and severe anaemia is when haemoglobin concentration less than 10.9g/dl and 7.0g/dl, respectively [11]. Globally, about 41.8% of pregnant women and 30.2% of non-pregnant women are anaemic, but in SSA, the proportion is higher with approximately 57% of women being anaemic [12,13]. The saving mothers report (2010-2013) states that 40% of maternal deaths in SA between 2010 and 2013 were related to anaemia [14]. Interventions to overcome or combat iron deficiency according to the South African National Department of Health Guidelines for Maternity Care (2015) recommend the oral intake of 200mg of ferrous sulphate tablets twice daily and 5mg of folic acid once daily [15].

Some recent studies conducted in Kwa-Zulu Natal, SA, demonstrated a much higher prevalence of anaemia in HIV positive pregnant women than in their HIV negative counterparts [6,8,16-22]. CD4 cell count remained a significant risk factor for anaemia among pregnant women pre and post-natally, after adjusting for age, ART regimen and gravidity [18]. The severity of the cases differed between studies, however most of the respondents presented with anaemia or moderate anaemia while the occurrence of severe anaemia was rare [8,16-22]. Anaemia during pregnancy can have negative maternal and neonatal outcomes such as increased risk of delivering low birth weight (LBW) infants as well as a higher risk of fetal anaemia [8,23]. In the mother, anaemia can cause decreased physical and mental capacity, reduced tolerance to infections and maternal mortality because of anaemic heart failure. In the context of HIV, iron deficiency and maternal anaemia are independent markers of disease progression and mortality [18,23-25]. Although studies to determine the incidence and predictors of anaemia in HIV infected patients receiving ART have been conducted at Themba Lethu Clinic, there is, however, limited data on anaemia in HIV infected pregnant women. This study is one of the few that have investigated maternal anaemia among the HIV infected pregnant women receiving treatment at the largest public sector HIV treatment site in SA.

## Methods

**Study design:** we analyzed secondary data of all HIV-positive women receiving ART at Themba Lethu Clinic, Helen Joseph Hospital in Johannesburg, South Africa. We included data of patients enrolled between 1^st^April 2004 (baseline) to 30^th^ April 2011 (end-line) and tracked anaemia among those patients seven months later. The Themba Lethu Clinic is the largest public sector ART site in the country [26].

**Study population:** our study included retrospective records for 236 HIV positive women aged 18-49 years on ART. Of these 118 were pregnant and 118 were non-pregnant HIV positive women. Patients included had the following parameters “baseline CD4 count, haemoglobin measurements and at least 7 months of follow-up time”. Both pregnant and non-pregnant study participants were offered similar first line ART regimens comprised of fixed dose combinations of stavudine (D4T), tenofovir (TDF), efavirenz (EFV), zidovudine (AZT), lamivudine (3TC) and neviropine (NVP). Specific triple therapy regimens included D4T/3TC/NVP; D4T/3TC/EFV; 3TC/TDF/EFV; 3TC/TDF/NVP; and AZT/3TC/NVP.

**Study variables:** the outcome variable was anaemia. This was defined as the haemoglobin concentration as per the Demographic and Health Survey (DHS) [27]. Guidelines “severe anaemia (<7.0g/dl), moderate anaemia (7.0 - 9.9g/dl), anaemia (10.0 - 10.9g/dl)”. This allowed us to have a more flexible definition that considers the pregnancy status of half of the study participants. We combined moderate and severe anaemia into one single category because there were very few patients classified as having severe anaemia; either at baseline (n=7) or at end-line (n=2). The analyses took into account the sociodemographic characteristics of the patients i.e. “age” (in complete years), pregnancy status (Y/N), education (no education, primary or just literate, secondary and beyond), smoking status (Y/N), alcohol consumption (Y/N) and employment status (employed/unemployed). Clinical and anthropometric measurements included body weight and height, which were used to compute a body mass index (BMI). We categorized BMI into the following using the Centers for Disease Control and Prevention (CDC) cut-off points: underweight: BMI <18.5, normal: BMI 18.5-24.5, overweight: BMI 25-29.9, obese: BMI ≥30. Furthermore, we included patients´ “CD4 count at baseline WHO stage of HIV disease at baseline and 7 months follow-up time after initiation of treatment”.

**Statistical analysis:** we performed data analysis using STATA 15. Patient´s demographics and clinical characteristics at baseline and end-line were described using percentages and frequencies for all categorical data. Means and standard deviation were used for all continuous variables. Given that our primary exposure was pregnancy and the two groups were balanced with respect to age and year of ART initiation, we also created a dichotomous variable for anaemia coded 1 for patients with any type of anaemia and 0 if otherwise. We then used the McNemar´s test to investigate the association between overall anaemia status (dichotomous) between the two data points (baseline and exit). For other categorical variables with more than 2 categories, we used Pearson Chi-square test to document any association between those characteristics and different levels of anaemia. An analysis of variance (one-way ANOVA) was used to test for equality of mean ‘CD4 count at baseline and end-line’ across the three levels of anaemia. A small proportion of patients had item missing data on CD4 count (n=7 at baseline and n=49 at end-line) and BMI (n=22 at baseline, n=36 at end-line). These item-missing values were replaced by the mean values in the series to reduce non-response bias. A sensitivity analysis was conducted with complete case analysis and we found no evidence that the mean imputation introduced a bias to the estimates. We used an ordered logistic regression model to predict the likelihood of these patients being in the higher versus the lower category of anaemia levels as a function of selected covariates, using pregnancy as the primary exposure variable. The final model excluded 10 patients with unknown status on history of alcohol use. Results from Brant test showed no violation of the proportional odds assumption.

**Ethical approval:** ethical clearance was obtained from Monash University Human Research Ethics Committee (certificate number 2016-0696).

## Results

At baseline, the two groups of HIV-positive female patients were similar in every aspect except for their age. Nearly 75% of the patients were in the age group (25-34 years); 80% and 68% of the pregnant and non-pregnant women respectively, being in this age category (Table 1). There were 143 HIV positive women classified as anaemic of which 56 had having moderate/severe anaemia and 87 had anaemia. Using McNemar test for marginal frequency, we found that the proportion of pregnant women classified as anaemic (anaemia, moderate/severe) at baseline (60.6%, n=143) was statistically different (p=0.001) from that of anaemic women at end-line (36.0%, n=85 patients). Of the 236 participants; 7(3.0%) were categorized as severe anaemia at baseline. These were combined with patients classified as having moderate anaemia and we found that more than half of the patients were either anaemic (36.9%) or had moderate/severe (23.7%) anaemia at baseline. Patients differed significantly at baseline with respect to the following characteristics: BMI categories (p=0. 01); WHO stage (p=0.001) and CD4 count (p-value: 0.001). For instance, at baseline the CD4 count of patients were relatively low among anaemic patients (mean: 122.7; sd: 95.9) whereas non-anemic patients had on average higher CD4 count (mean: 189.8; sd: 161.5). Anaemia was higher among underweight women and those in the normal BMI range as opposed to overweight and obese patients (Table 2).

**Table 1:** baseline characteristics of participants, by pregnancy status

Characteristics	Non-pregnant N=118 (n/N) %	Pregnant N=118 (n/N) %	p-value
**Age category**			
<25	12 (10.2)	14 (11.9)	0.008
25-34	80 (67.8)	95 (80.5)	
35-49	26 (22.0)	9 (7.6)	
**BMI category**			
Underweight	17 (14.5)	15 (12.7)	0.35
Normal	74 (63.3)	68 (57.6)	
Overweight	19 (16.2)	20 (17.0)	
Obese	7 (6.0)	15 (12.7)	
**Alcohol status**			
Non-consumer	102 (90.3)	101 (89.4)	0.83
Consumer	11 (9.7)	12 (10.6)	
WHO stage			
1	51 (43.2)	57 (48.3)	0.86
2	24 (20.4)	20 (17.0)	
3	32 (27.1)	31 (26.3)	
4	11 (9.3)	10 (8.4)	
**Education**			
No education	25 (21.2)	17 (14.4)	0.16
Primary or literate	7 (5.9)	14 (11.9)	
Secondary and beyond	86 (72.9)	87 (73.7)	
**Occupation**			
Disabled or unknown	8 (6.8)	5 (4.2)	0.48
Employed	52 (44.1)	47 (39.9)	
Unemployed	58 (49.1)	66 (55.9)	
**Smoking status**			
Non-smoker	112 (94.9)	111 (94.1)	0.78
Smokers	6 (5.1)	7 (5.9)	
CD4 count (mean, sd.)	142.26 (116.8)	156.72 (139.5)	0.39^t^

Note: p-value refer to Pearson Chi-square test, unless otherwise indicated; ^t^p-value refers to independent t-test

**Table 2:** baseline characteristics of the study participants

	Anemia status at baseline			
Characteristics	Severe/moderate anemia N=56 (n/N) %	Anemia N=87( n/N) %	No anemia N=93 (n/N) %	p-value
**Pregnancy status**				
Non-pregnant	30 (53.6)	42 (48.3)	46 (49.5)	0.82
Pregnant	26 (46.4)	45 (51.7)	47 (50.5)	
**Age category**				
<25	4 (7.1)	13 (15.0)	9 (9.7)	0.37
25 - 34	41 (73.2)	65 (74.7)	69 (74.2)	
35 - 49	11 (19.7)	9 (10.3)	15 (16.1)	
**BMI category**				
Underweight	15 (27.8)	12 (13.8)	5 (5.4)	0.01*^F^
Normal	31 (55.4)	56 (64.4)	55 (59.8)	
Overweight	6 (9.7)	13 (14.9)	20 (21.7)	
Obese	4 (7.1)	6 (6.9)	12 (13.1)	
**Alcohol status**				
Non-consumer	51 (92.7)	75 (91.5)	77 (86.5)	0.46^F^
Consumer^1^	4 (7.3)	7 (8.5)	12 (13.5)	
**WHO stage**				
1	14 (25.0)	41 (47.1)	53 (57.0)	0.001***
2	8 (14.3)	16 (18.4)	20 (21.5)	
3	25 (44.6)	24 (27.6)	14 (15.0)	
4	9 (16.1)	6 (6.9)	6 (6.5)	
**Education**				
No education	14 (25.0)	15 (17.2)	13 (14.0)	0.40
Primary or literate	4 (7.1)	10 (11.5)	7 (7.5)	
Secondary & beyond	38 (67.9)	62 (71.3)	73 (78.5)	
**Occupation**				
Disabled or unknown	1 (1.8)	4 (4.6)	8 (8.6)	0.49^F^
Employed	23 (41.1)	39 (44.8)	37 (39.8)	
Unemployed	32 (57.1)	44 (50.6)	48 (51.6)	
**Smoking status**				
Non-smoker	53 (94.6)	81 (93.1)	89 (95.7)	0.77^F^
Smokers	3 (5.4)	6 (6.9)	4 (4.3)	
CD4 count (mean, sd.)	124.1 (90.1)	122.7 (95.9)	189.8 (161.5)	0.001***^A^

Note: p-values from Pearson Chi-square test, unless otherwise indicated. ^F^: p-value from Fisher exact ^A^: p-value from one-way Anova assuming equal variance. ^†^p.10. ^*^p .05. ^**^p.01. ^***^p.001

At end-line (after seven months of treatment), the number of women with moderate/severe anaemia decreased significantly from 56 at baseline to 23. In addition, the number of HIV positive women with anaemia decreased from 87 at baseline to 62 seven months after starting treatment. Furthermore, the average CD4 count of women with moderate/severe anaemia increased from 124.1 to 287.3. However, across the three groups (non-anaemic, anaemia and moderate/severe anaemia) there was no statistically significant difference in CD4 count (Table 3). In the ordered logistic regression model, we found no association between anaemia status (aneamia or severely/moderately anaemic) and pregnancy status of the patients at baseline and seven months later. However, among patients with WHO stage 3 HIV disease at baseline, the odds of moderate/severe anaemia or anaemia versus no anaemia was 2.64 (95%: C.I: 1.35-5.15) compared to patients with WHO stage 1. Among patients with secondary/tertiary education, the odds of moderate/severe anaemia versus anaemia or no anaemia was 0.47 (95% C.I: 0.22-1.00) lower than patients with no education. At end-line, there was no statistically significant factor associated with anaemia of any grade (Table 4).

**Table 3:** end-line characteristics of study participants

Characteristics	Anemia Status at end-line			
	Severe/moderate N=23 (n/N) %	Anemia N=62 (n/N) %	No anemia N=151(n/N) %	p-value
Pregnancy status				
Non-pregnant	14 (60.9)	30 (48.4)	74 (49.0)	0.55
Pregnant	9 (39.1)	32 (51.6)	77 (51.0)	
Age category				
<25	2 (8.7)	10 (16.1)	14 (9.3)	0.69^F^
25 - 34	18 (78.3)	43 (69.4)	114 (75.5)	
35 - 49	3 (13.0)	9 (14.5)	23 (15.2)	
BMI category				
Underweight	7 (30.4)	10 (16.1)	15 (10.0)	0.23^F^
Normal	13 (56.5)	35 (56.)	94 (62.7)	
Overweight	2 (8.7)	12 (19.4)	25 (16.6)	
Obese	1 (4.4)	5 (8.1)	16 (10.7)	
Alcohol status				
Non-consumer	21 (95.5)	52 (89.7)	130 (89.0)	0.84^F^
Consumer^1^	1 (4.5)	6 (10.3)	16 (11.0)	
WHO stage				
1	9 (39.1)	30 (49.4)	69 (45.7)	0.63^F^
2	6 (26.1)	12 (19.3)	26 (17.2)	
3	5 (21.8)	13 (20.0)	45 (29.8)	
4	3 (13.0)	7 (11.3)	11 (7.3)	
Education				
No education	4 (17.3)	13 (21.0)	25 (16.6)	0.41^F^
Primary or literate	2 (8.7)	2 (3.2)	17 (11.2)	
Secondary and beyond	17 (74.0)	47 (75.8)	109 (72.2)	
Occupation				
Disabled or unknown	0 (0.00)	3 (4.8)	10 (6.6)	0.72
Employed	9 (39.1)	23 (37.1)	67 (44.4)	
Unemployed	14 (60.9)	36 (58.1)	74 (49.0)	
Smoking status				
Non-smoker	21 (91.3)	58 (93.6)	144 (95.4)	0.68
Smokers	2 (8.7)	4 (6.4)	7 (4.6)	
CD4 count (mean, sd.)	287.3 (89.0)	258.6 (120.5)	280.6 (150.4)	0.52

Note: p-values from Pearson Chi-square test, unless otherwise indicated. ^F^: p-value from Fisher exact ^A^: p-value from one-way Anova assuming equal variance. ^†^p.10. ^*^p .05. ^**^p.01. ^***^p.00, ^1^current or past

**Table 4:** ordered logistic regression model for prediction of anaemia (no-anaemia, mild anaemia, moderate/severe anaemia)

Characteristics	Baseline model	End-line model
	Moderate/severe & anemia vs. no anemia	moderate/severe & anemia vs. no anemia
**Pregnancy status**		
Non-pregnant	Ref	Ref
Pregnant	0.94 (0.56 - 1.61)	1.11 (0.60 - 2.06)
**Age category**		
<25	Ref	Ref
25 - 34	1.26 (0.55 - 2.88)	0.78 (0.34 - 1.70)
35 - 49	0.99 (0.35 - 2.79)	0.72 (0.25 - 2.08)
**BMI category**		
Underweight	Ref	Ref
Normal	0.43 (0.19 - 0.96) ^*^	0.54 (0.17 - 1.70)
Overweight	0.22 (0.08 - 0.60 ^**^	0.42 (0.14 - 1.32)
Obese	0.24 (0.08 - 0.76) ^*^	0.36 (0.10 - 1.33)
**Alcohol status**		
Non-consumer	Ref	Ref
Consumer	0.54 (0.21 - 1.40)	0.51 (0.18 - 1.47)
**WHO stage**		
1	Ref	Ref
2	1.13 (0.55 - 2.33)	1.78 (0.86 - 3.67)
3	2.64 (1.35 - 5.15) ^**^	0.86 (0.37 - 2.00)
4	2.54 (0.95 - 6.75)	1.94 (0.62 - 6.11)
**Education**		
No education	Ref	Ref
Primary or literate	0.51 (0.17 - 1.49)	0.40 (0.11 - 1.53)
Secondary and higher	0.47 (0.22 - 1.00) ^*^	0.76 (0.34 - 1.66) ^*^
**Occupation**		
Disabled or unknown	Ref	Ref
Employed	3.68 (0.72 - 18.85)	3.30 (0.38 - 28.87)
Unemployed	3.28 (0.65 - 16.63)	4.17 (0.48 - 35.80)
**Smoking status**		
Non-smoker	Ref	Ref
Smoker	1.60 (0.48 - 5.27)	1.95 (0.55 - 6.98)
CD4 Count (mean, sd.)	1.00 (0.995 - 1.00)	1.00 (1.00 - 1.001)

† p.10. ^*^p .05. ^**^p.01. ^***^p.001

## Discussion

The prevalence of anaemia was 60.6% at baseline and 36.0% after seven months of follow-up, showing a decline in the proportion of anaemic status with treatment. Previous studies have shown that prevalence of anaemia was higher among ART naive HIV positive pregnant women and declined once ART was initiated [27-29]. Such findings highlight the indirect positive effect of ART on anaemia among HIV positive pregnant women due to among other things immune recovery and improved general wellbeing. This highlights the need for continued scale up of ART coverage among HIV positive women in the population. We aimed to document the effect of pregnancy on anaemia among HIV patients. Using a dummy definition of anaemia (‘1’ for any level of anaemia and ‘0’ for no anaemia), we found no evidence that pregnancy was an aggravating factor for anaemia among study participants at baseline (OR: 0.94; 95% C.I: 0.56-1.61). At end-line, there is evidence that pregnant women were less likely to have higher levels of anaemia. However, this observed difference was not statistical significant (OR: 1.11; 95%: 0.60-2.06). Other previous studies have however reported pregnancy as a risk factor for clinical anaemia and that pregnant women had a 1-3 fold increased risk of developing anaemia than their non-pregnant counterparts [30]. Data suggests that ART had a positive indirect effect on anaemia among HIV positive patients. Even though our data could not establish an association between anaemia in HIV positive pregnant versus HIV positive non-pregnant women, the observed absence of a relationship might be due to existing differences in age distribution of our study population.

The study Banerjee *et al*. referenced above had a much younger sample of women and predominantly of the age group of 10-20 years was significantly associated with the development of anaemia in pregnant women [30]. The age range of our participants was (18 - 49 years) however, approximately 75% of the patients in their middle ages (25 - 34 years). The relationship between a woman's age and anaemia is widely reported and suggests that there is an association between anaemia and younger age particularly in adolescent girls and young women [31]. The risk of anaemia increases when adolescent girls and young women become pregnant [32]. Anaemia in this age group is often attributed to iron deficiency. Iron is essential in the development of a woman, especially during puberty, as this period of rapid growth often requires more iron to meet the physiological needs, coupled with blood loss during menstruation [33]. While it is well established that anaemia is a common disorder during pregnancy, our findings imply that pregnancy alone, as a determinant of anaemia is variable and that risk factors of anaemia are multifactorial. Therefore, a greater understanding of this association may play an important role in the management of clinical anaemia in pregnancy especially among those who are HIV infected. At baseline, our study showed that WHO stage, BMI category, education and CD4 count were significant predictors of anaemia. Patients with advanced HIV disease (WHO stage >2) were more likely to be in the higher category of anaemia compared with patients who did not have advanced HIV disease. However, after seven months of treatment, this difference was nonexistent suggesting that the observed difference in the likelihood of developing anaemia existed regardless of WHO stage, as long as the patient was on ART.

This is consistent with similar studies that suggest ART helps reverse clinical anaemia among HIV positive patients [33,34]. Other studies have suggested that anaemia is also a frequent complication among patients with low vitamin D status, iron deficiency, helminth infection, malaria infection and co-infection with Tuberculosis (TB) [35-38]. Although our study attributes the resolution of anaemia to ART use, we did not determine whether the presence of anaemia was a result of iron deficiency or of haematinic supplements in pregnancy. As such, we could not rule out the possibility of the positive effect of haematinic supplementation contributing to the resolution of anaemia among HIV positive pregnant women. Education was a key predictor of anaemia at baseline. The relationship between education and women´s health has widely been documented [39].

Education improves maternal and child health outcomes through different catalytic pathways. Similarly, we found that women with higher education were less likely to have anaemia at any stage of HIV disease compared with women with no or low education. However, as previously reported women in urban areas, with higher education attainment, were more likely to have anaemia [40]. Our findings suggest a different relationship, especially because our study setting was in an urban area. Better-educated women in society are more likely to be employed and be of a higher socio-economic status, have economic means to have regular meals, sustain a balanced diet and have better access to health care. Other previous studies have demonstrated the way in which anaemia interacts with socio-economic class and have reported higher prevalence of anaemia in patients of low ‘socio-economic status, educational level’, reduced meal frequency and residing in impoverished rural locations [41-43]. Our study had one main limitation. We did not assess the effect of other medications that the patients took on anaemia; such other medications might have had an effect on haemoglobin concentration or might have positively or otherwise influenced the patient´s compliance to taking haematinic supplementation.

## Conclusion

Our study has shown that ART use in HIV infected pregnant women with anaemia improves haemoglobin concentration regardless of baseline CD4 count or WHO stage of HIV disease within the first 7 months of ART usage. This supports the evidence that suggests that initiation and adherence to ART enhances the resolution of anaemia in HIV infected individuals regardless of the degree of immunosuppression at initiation. In view of the these findings it is important to: encourage universal access to ART among HIV positive patients, especially pregnant women; identify efforts to prevent anaemia in vulnerable people within the national HIV programs; have more frequent routine testing for anaemia in women especially those made vulnerable to the condition by HIV infection; enforce interventions and treatment efforts to prevent anaemia among those at higher risk by employing a multidisciplinary approach from a health care provider perspective including where possible, involving the services of a nutritionist.

### What is known about this topic

- Anaemia among women of childbearing age is a common condition in developing countries and complicates outcomes of pregnancy;
- The presence of anaemia among HIV infected individuals generally contributes to the progression of HIV infection to advanced disease.

### What this study adds

- Anaemia is a significant risk factor for poor health outcomes, especially among HIV positive pregnant women;
- The proportion of HIV positive women with anaemia decreased significantly, as such, ART access certainly shows to have a significant impact in reducing the intensity of anaemia in pregnancy;
- Monitoring HIV positive pregnant women is crucial to ensure that they have access to ART at an early stage.

## References

[1] Osungbade KO, Oladunjoye AO (2012). Anaemia in developing countries: burden and prospects of prevention and control.

[2] Rahman MM, Abe SK, Rahman MS, Kanda M, Narita S, Bilano B (2016). Maternal anaemia and risk of adverse birth and health outcomes in low-and middle-income countries: systematic review and meta-analysis. AJCN.

[3] Ndlovu Z, Chirwa T, Takuva S (2014). Incidence and predictors of recovery from anaemia within an HIV-infected South African cohort 2004-2010. Pan African Medical Journal.

[4] (2018). Human Sciences Research Council. The fifth South African national HIV prevalence, incidence, behaviour and communication survey, 2017, HIV impact assessment summary report. HSRC press Cape Town.

[5] Assefa M, Abegaz WE, Shewamare A, Medhin G, Belay M (2015). Prevalence and correlates of anaemia among HIV infected patients on highly active anti-retroviral therapy at Zewditu Memorial Hospital, Ethiopia. BMC Hematol.

[6] O´Brien ME, Kupka R, Msimanga GI, Saathoff E, Hunter DJ, Fawzi WW (2015). Anaemia is an independent predictor of mortality and immunologic progression of disease among women with HIV in Tanzania. J Acquir Immune Defic Syndr.

[7] Msuya SE, Hussein TH, Uriyo J, Sam NE, Pedersen BS (2011). Anaemia among pregnant women in northern Tanzania: prevalence, risk factors and effect on perinatal outcomes. Tanzan J Health Res.

[8] Tunkyi K, Moodley J (2016). Prevalence of anaemia in pregnancy in a regional health facility in South Africa. S Afr Med J.

[9] Ngene NC, Moodley J, Songca P, von Rahden R, Paruk F, Onyia CO (2013). Maternal and foetal outcomes of HIV-infected and non-infected pregnant women admitted to two intensive care units in Pietermaritzburg, South Africa. S Afr Med J.

[10] SShisana O, Rehle T, Simbayi LC, Zuma K, Jooste S, Zungu N (2014). South African national HIV prevalence, incidence and behaviour survey, 2012. Cape Town.

[11] WHO (2011). Haemoglobin concentrations for the diagnosis of anaemia and assessment of severity. Vitamin and Mineral Nutrition Information System.

[12] Ndukwu GU, Dienye PO (2012). Prevalence and socio-demographic factors associated with anaemia in pregnancy in a primary health center in Rivers State, Nigeria. Afr J Prim Health Care Fam Med.

[13] Alem M, Enawgaw B, Gelaw A, Kena T, Seid M, Olkeba Y (2013). Prevalence of anaemia and associated risk factors among pregnant women attending antenatal care in Azezo Health Center Gondar town, Northwest Ethiopia. J Interdiscipl Histopathol.

[14] National Department of Health (2013). Saving mothers 2010-2013: sixth report of confidential enquiries into maternal deaths in South Africa.

[15] National Department of Health, South Africa (2015). Guidelines for maternity care in South Africa: a manual for clinics, community health centres and district hospitals. Pretoria: NDOH.

[16] Ohihoin AG, Musa J, Sagay AS, Ujah IAO, Herbertson EC, Ocheke A (2014). Prevalence and determinants of anaemia among HIV positive pregnant women attending ante-natal clinic at the Jos University Teaching Hospital, Jos, North-central Nigeria. Br J of Med Med Res.

[17] Okoh DA, Iyalla C, Omunakwe H, Iwo-Amah RS, Nwabuko C (2016). A retrospective study of the prevalence of anaemia in pregnancy at booking in Niger Delta, Nigeria. J Ostet Gynaecol.

[18] Bodeau-livinec F, Briand V, Berger J, Xiong X, Massougbodji A, Day KP (2011). Maternal anaemia in Benin: prevalence, risk factors and association with low birth weight. Am J Trop Med Hyg.

[19] Nandlal V, Moodley D, Grobler A, Bagratee J, Maharaj NR, Richardson P (2014). Anaemia in pregnancy is associated with advanced HIV disease. PLoS One.

[20] Siteti MC, Namasaka SD, Ariya OP, Injete SD, Wanyonyi WA (2014). Anaemia in pregnancy: prevalence and possible risk factors in Kakamega County, Kenya. Sci J Pub Hlth.

[21] Melku M, Addis Z, Alem M, Enawgaw B (2014). Prevalence and predictors of maternal anaemia during pregnancy in Gondar, Northwest Ethiopia: an institutional based cross-sectional study. Anaemia.

[22] Ayenew F, Abere Y, Timerga G (2014). Pregnancy anaemia prevalence and associated factors among women attending ante natal care in Debre Berhan Health Institutions, Ethiopia. J Women´s Health Care.

[23] Laar AK, Grant FE, Addo Y, Soyiri I, Nkansah B, Abugri J (2013). Predictors of foetal anaemia and cord blood malaria parasitemia among new-borns of HIV-positive mothers. BMC Res Notes.

[24] Obse N, Mossie A, Gobena T (2013). Magnitude of anaemia and associated risk factors among pregnant women attending antenatal care in Shalla Woreda, West Arsi Zone, Oromia Region, Ethiopia. Ethiop J Health Sci.

[25] Olatunbosun OA, Abasiattai AM, Bassey EA, James RS, Ibanga G, Morgan A (2014). Prevalence of anaemia among pregnant women at booking in the University of Uyo Teaching Hospital, Uyo, Nigeria. BioMed Res Int.

[26] Fox MP, Maskew M, MacPphail AP, Long L, Brennan AT, Westreich D (2013). Cohort profile: the Themba Lethu Clinical cohort, Johannesburg, South Africa. Int J Epidemiol.

[27] National Department of Health (NDoH), Statistics South Africa (Stats SA), South African Medical Research Council (SAMRC) and ICF (2019). South Africa Demographic and Health Survey 2016. Pretoria, South Africa and Rockville, Maryland, USA.

[28] Tunkyi K, Moodley J (2017). Anaemia in pregnancy in a setting of high HIV prevalence rates. Southern African Journal of Infectious Diseases.

[29] Odhiambo C, Zeh C, Angira F, Opollo V, Akinyi B, Masaba R (2016). Anaemia in HIV-infected pregnant women receiving triple antiretroviral combination therapy for prevention of mother-to-child transmission: a secondary analysis of the Kisumu breastfeeding study (KiBS). Trop Med Int Health.

[30] Oladeinde BH, Omoregie R, Olley M, Anunibe A (2011). Prevalence of HIV and anaemia among pregnant women. N Am J Med Sci.

[31] Banerjee B, Pandey GK, Dutt D, Sengupta B, Mondal M, Deb S (2009). Teenage pregnancy: a socially inflicted health hazard. Indian J Community Med.

[32] Intiful FD, Wiredu EK, Asare GA, Asante M, Adjei DN (2016). Anaemia in pregnant adolescent girls with malaria and practicing pica. Pan African Medical Journal.

[33] Li N, Sando MM, Spiegelman D, Hertzmark E, Liu E, Sando D (2016). Antiretroviral therapy in relation to birth outcomes among HIV-infected women: a cohort study. J Infect Dis.

[34] Takuva S, Maskew M, Brennan AT, Sanne I, Macphail AP, Fox MP (2013). Anaemia among HIV-infected patients initiating antiretroviral therapy in South Africa: improvement in haemoglobin regardless of degree of immunosuppression and the initiating ART regimen. J Trop Med.

[35] Isanaka S, Mugusi F, Urassa W, Willett WC, Bosch RJ, Villamor E (2012). Iron deficiency and anaemia predict mortality in patients with tuberculosis. J Nutr.

[36] Hoque M, Hoque E, Kader SB (2009). Risk factors for anaemia in pregnancy in rural KwaZulu-Natal, South Africa: implication for health education and health promotion. South African Family Practice.

[37] Ouédraogo S, Koura GK, Accrombessi MMK, Bodeau-Livinec F, Massougbodji A, Cot M (2012). Maternal anaemia at first antenatal visit: prevalence and risk factors in a malaria-endemic area in Benin. Am J Trop Med Hyg.

[38] Adam I, Ibrahim Y, Elhardello O (2018). Prevalence, types and determinants of anaemia among pregnant women in Sudan: a systematic review and meta-analysis. BMC Hematol.

[39] Onarheim KH, Iversen JH, Bloom DE (2016). Economic benefits of investing in women´s health: a systematic review. PloS One.

[40] Adamu AL, Crampin A, Kayuni N, Amberbir A, Koole O, Phiri A (2017). Prevalence and risk factors for anaemia severity and type in Malawian men and women: urban and rural differences. Popul Health Metr.

[41] Lebso M, Anato A, Loha E (2017). Prevalence of anaemia and associated factors among pregnant women in Southern Ethiopia: a community based cross-sectional study. PLoS One.

[42] Gebre A, Mulugeta A (2015). Prevalence of anaemia and associated factors among pregnant women in north western Zone of Tigray, Northern Ethiopia: a cross-sectional study. J Nutr Metab.

[43] Stephen G, Mgongo M, Hussein Hashim T, Katanga J, Stray-Pedersen B, Msuya SE (2018). Anaemia in pregnancy: prevalence, risk factors and adverse perinatal outcomes in Northern Tanzania. Anaemia.

